# The Potential of Red Blood Cells in Regenerative Medicine: A Paradigm Shift in Cellular Therapy

**DOI:** 10.3390/cells14110797

**Published:** 2025-05-29

**Authors:** Fábio Ramos Costa, Joseph Purita, Ansar Mahmood, Rubens Martins, Bruno Costa, Bruno Lima Rodrigues, Stephany Cares Huber, Gabriel Silva Santos, Luyddy Pires, Gabriel Azzini, André Kruel, José Fábio Lana

**Affiliations:** 1 Department of Orthopedics, FC Sports Traumatology, Salvador 40296-210, BA, Brazil; 2 Orthopedics, PUR-FORM, Boca Raton, FL 33432, USA; jpurita@aol.com; 3 Trauma and Orthopaedics Department, University Hospitals Birmingham NHS Foundation Trust, Queen Elizabeth Hospital, Birmingham B15 2GW, UK; ansar.mahmood@uhb.nhs.uk; 4 Medical School, Tiradentes University Center, Maceió 57038-000, AL, Brazil; rubensdeandrade@hotmail.com; 5 Medical School, Zarns College, Salvador 41720-200, BA, Brazil; fabiocosta7113@gmail.com; 6 Department of Orthopedics, Brazilian Institute of Regenerative Medicine (BIRM), Indaiatuba 13334-170, SP, Brazil; brunolr.ioc@gmail.com (B.L.R.); stephany_huber@yahoo.com.br (S.C.H.); luyddypires@gmail.com (L.P.); drgabriel.azzini@gmail.com (G.A.); kruel.andre@gmail.com (A.K.); josefabiolana@gmail.com (J.F.L.); 7 Regenerative Medicine, Orthoregen International Course, Indaiatuba 13334-170, SP, Brazil; 8 Medical School, Max Planck University Center (UniMAX), Indaiatuba 13343-060, SP, Brazil; 9 Clinical Research, Anna Vitória Lana Institute (IAVL), Indaiatuba 13334-170, SP, Brazil; 10 Medical School, Jaguariúna University Center (UniFAJ), Jaguariúna 13911-094, SP, Brazil

**Keywords:** red blood cells, regenerative medicine, extracellular vesicles, immunomodulation, tissue engineering

## Abstract

Red blood cells (RBCs) have traditionally been excluded from orthobiologic formulations due to inflammation, oxidative stress, and hemolysis concerns. However, emerging evidence suggests that RBCs may play an active role in regenerative medicine, contributing to immune modulation, vascular support, and oxidative balance. Their interactions with macrophages, involvement in nitric oxide signaling, and release of extracellular vesicles suggest they may influence tissue repair more than previously assumed. Despite these potential benefits, RBC retention in orthobiologic preparations like platelet-rich plasma (PRP) and bone marrow aspirate concentrate (BMAC) remains controversial, with most protocols favoring their removal in the absence of robust translational clinical data. This review explores the biological functions of RBCs in regenerative medicine, their potential contributions to PRP and BMAC, and the challenges associated with their inclusion. While concerns about hemolysis and inflammation persist, controlled studies are needed to determine whether selective RBC retention could enhance musculoskeletal healing in some scenarios. Future research should focus on optimizing RBC processing techniques and evaluating their impact on clinical applications. Addressing these gaps will clarify whether RBCs represent an overlooked but valuable component in regenerative therapies or their exclusion remains justified.

## 1. Introduction

Regenerative medicine has transformed the treatment of musculoskeletal (MSK) disorders by introducing biologically active therapies that enhance tissue repair and modulate inflammation [1]. Orthobiologic formulations, including platelet-rich plasma (PRP) and bone marrow aspirate concentrate (BMAC), have gained widespread use in orthopedics due to their capacity to deliver growth factors, cytokines, and progenitor cells to injured tissues [2]. For the purposes of this review, we define orthobiologics as biologically derived substances that are used to stimulate musculoskeletal tissue repair through biochemical, cellular, or immunomodulatory mechanisms [3]. This includes established agents such as PRP and BMAC, as well as emerging components like red blood cell-derived extracellular vesicles, which may exert regenerative effects through paracrine signaling.

While much attention has been directed toward platelets, leukocytes, and mesenchymal stem cells (MSCs) in these formulations, RBCs have been mainly overlooked in the regenerative medicine paradigm. To contextualize the regenerative relevance of RBCs, Table 1 presents a comparative overview of major hematologic and regenerative cell types used in orthobiologic therapies, including their key functions and research status. Traditionally considered passive oxygen transporters, RBCs are routinely removed from orthobiologic preparations based on inflammation, oxidative stress, and hemolysis concerns [4]. However, emerging evidence suggests that RBCs may possess immunomodulatory, angiogenic, and regenerative properties, although their use remains controversial due to reports of cytotoxicity in intra-articular environments [5,6,7,8,9,10].

**Table 1:** Comparison of regenerative hematological components.

Cell Type	Primary Function	Regenerative Properties	Potential Applications	References
Red Blood Cells (RBCs)	Oxygen Transport	Extracellular vesicles (EVs), immunomodulation, oxygenation	Emerging translational research for regenerative uses	[11]
Mesenchymal Stem Cells (MSCs)	Tissue Regeneration	Differentiation into various cell types, paracrine signaling	Widely used in orthopedic, cardiac, and neurological treatments	[12]
Platelets	Clot Formation & Healing	Growth factor release, immune modulation	PRP widely used in wound healing, orthopedics	[13]
Macrophages	Immune Regulation	Phagocytosis, cytokine secretion, tissue repair	Experimental therapies in chronic inflammation, tissue healing	[12]

Despite being the most abundant cellular component in blood, RBCs have received minimal attention in the context of tissue regeneration. The prevailing assumption is that their presence in regenerative therapies is either redundant or detrimental due to their potential to release free hemoglobin and iron, which can drive oxidative damage [4,14]. Consequently, the standard approach in orthobiologic preparations such as PRP and BMAC is to separate and discard RBCs while leaving behind platelet-rich and stem cell-rich fractions [15]. However, this assumption has been challenged by recent findings which indicate that RBCs may contribute to immune modulation, extracellular vesicle signaling, and microenvironmental regulation, all of which are critical in tissue healing [11,16].

RBCs are central in maintaining tissue oxygenation and metabolic homeostasis, but their functions extend beyond simple oxygen transport. They have also been implicated in vascular function, immune signaling, and extracellular vesicle release—mechanisms that are relevant to tissue repair [17,18]. In particular, red blood cell-derived extracellular vesicles (RBCEVs) have been identified as carriers of bioactive lipids, proteins, and microRNAs (mi-RNAs), which may influence tissue repair and inflammatory pathways [19]. The regenerative potential of these vesicles has been explored in other fields, including cardiovascular and neurological research, yet their role in MSK disorders remains largely unexamined [20,21].

RBCs interact with monocyte/macrophage system components, which play a role in shifting macrophages toward an anti-inflammatory (M2) phenotype. This polarization is essential for resolving inflammation and promoting tissue remodeling [22,23], particularly in degenerative conditions such as osteoarthritis and tendinopathy [24]. RBC membranes also expose phosphatidylserine (PS), a key molecule that modulates immune tolerance and apoptotic cell clearance, which further underscores their potential role in regenerative medicine [25]. Heme oxygenase-1 (HO-1), an enzyme that is upregulated in response to RBC breakdown, has been shown to be involved in cytoprotective, angiogenic, and anti-inflammatory functions [26]. These mechanisms suggest that RBCs may serve an active role in tissue healing rather than merely being inflammatory byproducts.

Orthobiologic therapies, particularly PRP and BMAC, have become widely used in the treatment of joint disorders such as osteoarthritis, tendinopathy, and non-union fractures [27]. These therapies deliver autologous growth factors and cellular components to stimulate healing [15,28]. While platelets are central to these formulations due to their high concentration of growth factors such as platelet-derived growth factor (PDGF) and transforming growth factor-beta (TGF-β), excluding RBCs from PRP and BMAC has become a widely adopted standard, primarily due to on early observations linking the presence of RBCs to oxidative stress, inflammation, and cytotoxicity in joint environments. Given that PRP and BMAC are inherently hematological products, a deeper investigation into the role of RBCs within these formulations is warranted.

Few studies have systematically examined the potential benefits of retaining RBCs in regenerative formulations for orthopedic applications, and this oversight represents a significant gap in the literature. While RBCs are commonly removed due to concerns about oxidative stress, their angiogenic and immunoregulatory effects may provide previously unrecognized therapeutic advantages. Given the growing interest in optimizing regenerative approaches for MSK disorders, exploring the potential inclusion or modulation of RBCs in orthobiologic therapies represents an essential and timely research question.

This review evaluates the potential regenerative roles of RBCs in treating joint disorders. By analyzing the existing literature on RBC biology, orthobiologic formulations, and the emerging role of RBCEVs, we aim to challenge conventional perspectives and highlight a novel approach to regenerative medicine. Understanding how RBCs influence inflammation, tissue repair, and cellular signaling may inform future refinements in orthobiologic formulations. However, these hypotheses must be balanced against evidence of potential cytotoxic effects, which have been demonstrated particularly in synovial applications.

## 2. Biological Functions of Red Blood Cells in Regenerative Medicine

RBCs are the most abundant cellular component in human blood, and are traditionally recognized for their role in oxygen transport [29]. Beyond their well-established function in respiratory gas exchange, RBCs possess immunomodulatory, metabolic, and extracellular signaling properties that are increasingly being explored in various medical fields [5,6,7,8]. While most regenerative medicine research has focused on platelets, leukocytes, and MSCs, recent findings suggest that RBCs contribute to tissue repair, inflammation resolution, and vascular support [5,6,7,8]. These functions position RBCs as potentially valuable yet underexplored cellular mediators in regenerative medicine.

The primary function of RBCs is to facilitate oxygen transport from the lungs to peripheral tissues and remove carbon dioxide, a metabolic byproduct [29]. However, their biological roles extend beyond this, with evidence supporting their influence on vascular regulation, oxidative stress balance, and intercellular communication. RBCs regulate the vascular tone through NO metabolism, influencing angiogenesis and blood perfusion [18]. They carry and modulate NO bioavailability, contributing to vasodilation and blood flow regulation—critical factors in tissue healing and regenerative processes [18]. RBCs are also key regulators of oxidative stress, primarily through their glutathione system and enzymatic antioxidants, which include superoxide dismutase and catalase [4]. Since oxidative stress is a major driver of joint tissue degradation and chronic inflammation, RBCs may be essential in modulating these pathways, potentially providing additional antioxidant support in regenerative applications.

Contrary to the traditional assumption that RBCs contribute to inflammation, recent evidence suggests that they actively participate in immune regulation. Their interactions with the immune system occur through multiple mechanisms, including macrophage polarization and inflammation resolution [22,24]. RBCs influence macrophage behavior, shifting them toward the M2 phenotype, which is essential for cartilage protection and tendon healing [30,31]. In osteochondral injuries, it is plausible that RBCs contribute to modulating chronic low-grade inflammation, potentially through mechanisms such as macrophage polarization [32,33], although this remains to be directly demonstrated. Importantly, this hypothesis refers to the presence of RBCs in small, controlled concentrations, not in volumes that would disrupt the cellular balance or provoke adverse effects.

Furthermore, RBCs also express the Duffy antigen receptor for chemokines (DARC), a regulatory receptor for inflammatory chemokines [34]. By binding and sequestering pro-inflammatory cytokines, such as interleukin (IL)-8 and RANTES (regulated on activation, normal T-cell expressed and secreted), RBCs may play a role in reducing systemic inflammation and contributing to a regenerative microenvironment [34,35].

In addition to their metabolic and immunomodulatory functions, RBCs contribute to extracellular vesicle-mediated tissue repair. While platelet- and MSC-derived extracellular vesicles have been extensively studied, RBCEVs remain largely unexplored despite their ability to influence angiogenesis, inflammation, and oxidative stress [11,21]. RBCEVs contain diverse bioactive molecules, including miRNAs and siRNAs (small interfering RNAs), that regulate gene expression in target cells, and potentially influence MSK structures [19,36,37]. RBCEV membrane proteins influence endothelial function and blood vessel remodeling, supporting angiogenesis in tissue engineering applications, while lipid mediators modulate inflammatory responses and contribute to immune regulation [38]. Given that extracellular vesicles are already being explored as therapeutic agents, further research into RBCEV-based regenerative approaches could open new frontiers in MSK treatment.

Despite their biochemical and immunological contributions, RBCs remain routinely removed from PRP, BMAC, and other orthobiologic preparations. This exclusion is based on historical assumptions that RBCs are inflammatory or redundant in regenerative applications. However, considering their influence on immune modulation, angiogenesis, oxidative stress regulation, and extracellular vesicle communication, it is becoming increasingly clear that RBCs warrant further investigation in regenerative medicine.

## 3. Current Use of Orthobiologic Formulations in Joint Disorders

Orthobiologic therapies have become an integral part of modern regenerative medicine, particularly in treating MSK disorders such as osteoarthritis, tendinitis, and non-union fractures [27,39,40,41]. These therapies, derived from autologous or allogeneic biological sources, aim to enhance tissue healing, modulate inflammation, and restore functional integrity in damaged joints, tendons, and bones [15,42,43,44]. Among the most widely utilized orthobiologic formulations are PRP and BMAC, which are hematological products that rely on cellular and molecular components of blood to exert regenerative effects [28,45]. Despite significant progress in refining these therapies, the inclusion or exclusion of RBCs in these formulations is still up for debate, with most clinical protocols favoring RBC removal based on inflammation, cytotoxicity, and oxidative stress concerns. However, given the emerging evidence regarding the potential biological benefits of RBCs, it is important to reassess their role in regenerative medicine and to determine whether their limited and selective inclusion may enhance the efficacy of PRP and BMAC in joint disorders.

### 3.1. PRP and RBC Exclusion

PRP is one of the most extensively studied and utilized orthobiologic treatments for joint and soft tissue injuries. It is prepared by centrifuging whole blood to concentrate platelets that are rich in growth factors such as PDGF, vascular endothelial growth factor (VEGF), TGF-β, and insulin-like growth factor-1 (IGF-1) [15]. These bioactive molecules promote angiogenesis, collagen synthesis, and chondroprotection, making PRP a preferred option for treating degenerative joint conditions and chronic tendinopathies [15].

A vital aspect of PRP formulations is the presence or absence of RBCs during their processing [46]. Traditional protocols favor leukocyte-rich or leukocyte-poor PRP preparations, but almost all techniques exclude RBCs [15,41,45], primarily due to concerns about hemolysis, oxidative stress, and inflammation [10]. Indeed, free hemoglobin has been associated with inflammatory responses in the synovial environment, leading to fears that RBC contamination may exacerbate joint inflammation rather than contribute to healing [10].

However, the complete removal of RBCs from PRP may be an oversimplification of their biological role. Trace amounts of RBCs could exert immunoregulatory and antioxidant effects, which could contribute positively to tissue repair. In this context, the MARSPILL classification proposed by Lana et al. [46] defines “poor” RBC content as a ~15-fold reduction compared to baseline levels, which approximately corresponds to less than 5–7% of the original RBC volume in standard blood. This framework allows physicians to operationally define what constitutes a “low” or “trace” RBC presence in PRP formulations, distinguishing it from excessive levels (>10–15%), which have been associated with pro-inflammatory or cytotoxic responses.

RBCs possess high intracellular concentrations of glutathione, a key antioxidant that is involved in maintaining redox balance. Upon RBC activation or lysis, glutathione may be released into the extracellular environment, where it could contribute to oxidative stress modulation by neutralizing reactive oxygen species and supporting local antioxidant defenses [47]. While excessive RBC content has indeed been associated with pro-inflammatory responses and oxidative damage, we anticipate that the therapeutic efficacy of BMAC and PRP will not be affected by the presence of a minimal and well-controlled amount of RBCs relative to other PRP components. This is especially relevant when considering variations in PRP preparation methods. Handmade systems often retain trace amounts of RBCs, whereas automated commercial kits are generally designed to minimize the RBC content [46]. Given the potential immunoregulatory and vascular contributions of RBCs at low concentrations, further investigation is warranted to determine whether their selective retention in small quantities could enhance regenerative outcomes without introducing adverse effects.

### 3.2. BMAC and RBCs

BMAC is another widely used orthobiologic therapy that contains MSCs, hematopoietic progenitor cells, platelets, and a variety of bioactive molecules [28]. MSCs derived from BMAC have demonstrated chondroprotective, osteogenic, and immunomodulatory effects, making BMAC an attractive treatment option for cartilage defects, osteoarthritis, and fracture non-union [48,49] conditions.

Unlike PRP, which is primarily platelet-centric, BMAC is valued for its stem cell content, particularly its content of bone marrow-derived MSCs that contribute to cartilage and bone regeneration [28,50]. However, BMAC preparations also contain RBCs, and just like PRP, clinical protocols typically recommend their removal. RBC depletion from BMAC is performed due to concerns related to cellular debris, heme toxicity, and potential interference with MSC and chondrocyte function [51,52]. Nevertheless, the literature has not critically examined the exclusion of RBCs from BMAC. It is well established that the biological effects mediated by MSCs are largely attributed to paracrine mechanisms, including the secretion of cytokines, growth factors, chemokines, and extracellular vesicles (EVs) [53]. However, it remains unclear whether controlled RBC retention in small concentrations could enhance the regenerative impact of BMAC through the previously described mechanisms, which include vascular and intercellular support. Interestingly, some bone marrow aspiration systems, such as the Marrow Cellutions™ device, are designed to use unprocessed aspirate without centrifugation or RBC removal. These systems retain the full cellular content of the marrow aspirate, including red blood cells, representing an alternative approach to conventional BMAC processing.

## 4. Potential Benefits of RBCs in Orthobiologic Therapies

One of the most significant potential benefits of RBCs in regenerative medicine lies in their ability to modulate immune responses [7]. Macrophage polarization plays a central role in tissue healing, with pro-inflammatory (M1) macrophages dominating the early response to injury, while anti-inflammatory (M2) macrophages contribute to tissue repair and matrix remodeling [54]. RBCs interact with macrophages and have been shown to promote M2 polarization, through which they support a regenerative microenvironment [32].

Additionally, by sequestering cytokines such as IL-8 and RANTES, RBCs may dampen chronic inflammation in degenerative joint conditions [34,55]. This is particularly relevant in osteoarthritis, where persistent low-grade inflammation accelerates cartilage degradation and joint dysfunction [56]. The controlled retention of RBCs in orthobiologic preparations may help mitigate this inflammatory response, promoting immune balance and tissue repair.

RBC membranes also expose PS under certain conditions, facilitating apoptotic cell clearance and macrophage-driven inflammation resolution [25,33]. PS-exposing RBCs have been linked to the recruitment of regulatory immune cells that contribute to joint homeostasis and synovial integrity [57]. This function may be particularly relevant in conditions such as tendinitis and bone fractures, where dysregulated inflammatory responses impair healing and prolong recovery [58,59].

### 4.1. Angiogenesis and Vascular Homeostasis

Oxygenation and vascularization are critical determinants of tissue regeneration, particularly in the avascular or hypovascular environments found in cartilage, tendons, and some bone defects [60]. RBCs play a pivotal role in vascular regulation through their NO metabolism and delivery, which influences endothelial function, angiogenesis, and local tissue perfusion [17]. RBCs help regulate NO bioavailability, a key factor in capillary expansion and new vessel formation [17]. Given that orthobiologic therapies are often used in poorly vascularized tissues [61], such as the intra-articular space [62], RBCs may enhance oxygen distribution and nutrient exchange in these tissues [63,64], improving the metabolic conditions for repair.

RBCEVs have also been identified as carriers of angiogenic factors that influence vascular remodeling [21,65]. While platelet-derived EVs are known for their pro-angiogenic effects, the potential of RBCEVs to promote capillary network formation and endothelial repair remains largely unexplored. Given the importance of vascular support in musculoskeletal healing, this area represents a promising avenue for future research.

### 4.2. Oxidative Stress Regulation and Cytoprotective Pathways

**Figure 1:**
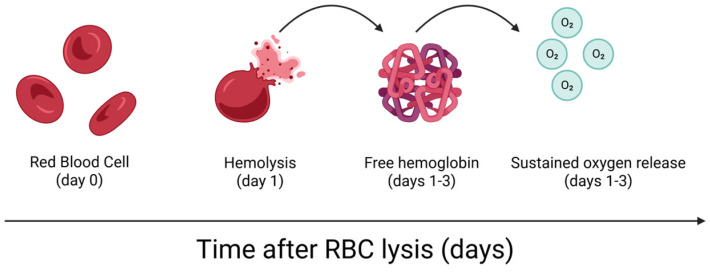
Temporal Dynamics of RBC Hemolysis and Oxygen Release. Temporal sequence of RBC hemolysis and subsequent sustained oxygen release. Following RBC lysis, free hemoglobin is released into the extracellular environment, contributing to oxidative stress and microenvironmental alterations through sustained oxygen release over several days. This process may influence the regenerative potential and inflammatory dynamics of orthobiologic preparations.

HO-1 activity triggered by RBC breakdown yields cytoprotective byproducts such as CO and biliverdin, supporting antioxidant defenses [66]. CO exerts anti-inflammatory and vasodilatory effects, while biliverdin and bilirubin are potent antioxidants [66]. Collectively, they may protect joint tissues from oxidative stress. The ability of HO-1 pathways to mitigate oxidative stress and support cell function suggests that the presence of RBCs in controlled conditions may provide protective benefits rather than harm. Furthermore, RBCs contain antioxidant systems, including glutathione, superoxide dismutase, and catalase, which help to neutralize ROS [67]. While these mechanisms suggest a potential for reducing oxidative tissue damage, such benefits remain hypothetical and must be considered in parallel with data showing that RBCs may also exacerbate oxidative stress in certain conditions [9,10,68]. Figure 1 illustrates the temporal sequence of RBC hemolysis and the subsequent sustained oxygen release via free hemoglobin, which may contribute to oxidative stress and microenvironmental signaling. Instead of complete RBC removal, optimizing the ratio of RBCs in PRP and BMAC could leverage these antioxidant functions while minimizing the hemolytic risk.

### 4.3. RBC-Derived Extracellular Vesicles in Cartilage and Tendon Healing

The role of extracellular vesicles in regenerative medicine has received growing attention. MSC- and platelet-derived EVs are extensively studied for their ability to deliver growth factors, regulate inflammation, and promote tissue regeneration [69]. While platelet- and MSC-derived EVs are actively secreted through complex intracellular machinery, RBCs lack nuclei and most organelles, which limits their capacity for active secretion. Instead, RBCEVs are primarily formed through passive membrane budding in response to cellular aging, mechanical stress, or oxidative conditions [11]. Although less studied in musculoskeletal applications, RBCEVs have shown regenerative potential that is comparable to that of MSC- and platelet-derived EVs [20,36]. These vesicles carry miRNAs, membrane proteins, and lipid mediators that may influence tissue remodeling and matrix homeostasis [21,32,70]. Their signaling pathways have been associated with endothelial repair, immunoregulation, and oxidative balance, which supports their potential as regenerative mediators [21,25,32,33,57,70].

## 5. Challenges, Controversies, and Future Directions

The integration of RBCs into orthobiologic therapies remains a subject of debate, with several biological and clinical uncertainties needing resolution before they are widely adopted. While recent findings highlight the potential benefits of RBCs in immune modulation, vascular homeostasis, and extracellular signaling, their role in tissue regeneration is not yet fully understood. The most significant challenges lie in concerns regarding hemolysis, oxidative stress, and inflammatory responses, which have historically led to their exclusion from PRP and BMAC. While early studies have reported potential cytotoxic effects associated with RBCs, the widespread exclusion of these cells from orthobiologic formulations has often preceded definitive experimental validation, particularly regarding their impact at low concentrations.

One of the primary challenges is the risk of hemolysis, where RBC degradation releases free hemoglobin, heme, and iron. These byproducts have been implicated in oxidative tissue damage and synovial inflammation, particularly in conditions that involve intra-articular bleeding. The presence of excessive iron can catalyze ROS formation, exacerbating oxidative stress within joint tissues. This has reinforced the assumption that RBCs may contribute to tissue degradation rather than supporting regenerative processes. These concerns are supported by in vitro studies which demonstrated that RBC-containing formulations may increase synoviocyte cytotoxicity and inflammatory cytokine release [9,10]. Also, the release of macrophage migration inhibitory factor (MIF) from RBCs has been proposed as a key contributor to inflammatory signaling within joint environments. MIF is known to stimulate the release of cytokines such as TNF-α, IL-1β, and IL-6, matrix metalloproteinase activation, and oxidative stress, and thereby amplify tissue degradation processes. These mechanisms were extensively reviewed by Everts et al. [71], who emphasized the potential cytotoxicity of RBCs in PRP and BMAC formulations through pathways involving MIF, free hemoglobin, and eryptosis.

However, not all RBC-derived metabolites exert harmful effects. The enzymatic activity of HO-1, which is upregulated in response to RBC breakdown, generates bioactive molecules with cytoprotective and anti-inflammatory properties. The balance between these opposing effects remains unclear, and it is not yet known whether controlled RBC retention at specific concentrations could shift the response toward a net regenerative effect. Future studies must define the threshold at which RBC inclusion enhances therapeutic efficacy without increasing the oxidative stress burden.

Another unresolved issue is the role of RBCs in inflammation. While RBC accumulation in pathological states has been associated with inflammatory responses, this does not necessarily translate to the controlled inclusion of RBCs in regenerative formulations. Their roles in cytokine modulation and apoptotic clearance may support immune resolution rather than promote inflammation. However, the extent to which these effects occur in PRP or BMAC remains unknown. More targeted research is needed to evaluate how RBCs interact with immune cells in the intra-articular space and whether their presence alters inflammatory dynamics in a way that promotes tissue repair. The potential for RBCs to exhibit both pro- and anti-inflammatory properties under different conditions further complicates this discussion, reinforcing the need for studies that assess their biological effects within defined regenerative environments.

A significant limitation in assessing the function of RBCs in regenerative medicine is the lack of standardized protocols for their processing and inclusion. Current methodologies favor RBC depletion without investigation into whether the selective retention of specific RBC subsets could improve clinical outcomes. There is still no consensus regarding the ideal RBC concentration for orthobiologic products or whether RBC subpopulations differ in their regenerative potential. While no standardized RBC thresholds currently exist for PRP or BMAC formulations, the available data suggest a concentration-dependent effect on cytotoxicity. For example, Braun et al. [9] reported that leukocyte-rich PRP contained 3.8 million RBCs per microliter, compared to just 0.09 million RBCs per microliter in leukocyte-poor PRP, with higher RBC counts correlating with increased synoviocyte death and inflammatory mediator release. Incorporating such quantitative parameters in future studies may help to define safe RBC concentration ranges tailored to different clinical indications and tissue environments. It is also important to consider that the biological behavior of RBCs may differ depending on their physical state and the local microenvironment. Intact RBCs may support homeostasis through antioxidant and signaling mechanisms, while lysed RBCs are more likely to release free hemoglobin and iron, promoting oxidative stress. Moreover, intra-articular tissues such as synovium appear to be more vulnerable to RBC-induced inflammation than relatively avascular environments like tendons, where the presence of RBCs may be less disruptive. These context-specific differences underscore the need for tissue-targeted studies to evaluate the safety and efficacy of RBC-containing formulations.

Additionally, the influence of RBCs on other cellular components in orthobiologic formulations remains poorly characterized. Their interactions with platelets, growth factors, and mesenchymal stem cells may play a crucial role in determining the overall efficacy of the therapy. Without a clear understanding of these interactions, the effects of RBC retention remain speculative. Developing precise techniques for processing RBCs, minimizing their hemolysis, and optimizing their incorporation into orthobiologic therapies will be essential for determining their true potential.

Despite growing interest in the potential of RBCs in regenerative medicine, clinical validation remains lacking. Most PRP and BMAC studies have been conducted using RBC-depleted formulations, presenting no clinical data on RBC retention. Without comparative trials, it isnot easy to assess whether RBC-containing preparations provide superior or equivalent outcomes compared to conventional RBC-depleted formulations. Future research should prioritize clinical studies that evaluate functional improvements, pain reduction, and structural changes in patients receiving RBC-containing PRP or BMAC. Prospective trials should also assess the long-term safety of RBCs, ensuring that any regenerative benefits associated with RBCs are not accompanied by unintended adverse effects such as excessive oxidative stress or sustained inflammation. Additionally, patient-specific factors must be considered, as certain musculoskeletal conditions may respond more favorably to RBC-enriched formulations than others.

## 6. Conclusions

Red blood cells have long been excluded from regenerative therapies, yet growing interest in their biological functions calls for a re-evaluation of this practice. Addressing these challenges will determine whether RBCs can transition from being viewed as contaminants to valuable therapeutic components in regenerative medicine. The growing recognition of their immunoregulatory and angiogenic properties suggests that their exclusion may have been premature. However, without further research into optimal processing techniques, their mechanisms of action, and their clinical efficacy, RBCs will remain controversial in regenerative medicine. Future studies should focus on defining their precise biological functions, optimizing their integration into existing orthobiologic therapies, and establishing safety parameters for their inclusion in clinical practice. RBCs can only be accurately positioned as potential mediators of musculoskeletal regeneration through rigorous investigation of their relationship to the existing complex milieu seen in orthobiologics. In addition to clarifying the role of intact RBCs, future investigations may also isolate RBCEVs as independent therapeutic agents, which could potentially allow for their use in regenerative applications without the risks associated with whole-cell inclusion. Furthermore, comparative trials that evaluate RBC-enriched versus RBC-depleted formulations in non-articular settings, such as tendons or fascia, may help define tissue-specific safety profiles and therapeutic windows. These directions may assist in optimizing the design and personalization of orthobiologic therapies.

## Data Availability

Not applicable.
